# AngII-induced glomerular mesangial cell proliferation inhibited by losartan via changes in intracellular calcium ion concentration

**DOI:** 10.1007/s10238-013-0232-y

**Published:** 2013-03-05

**Authors:** Guoying Qiu, Zequan Ji

**Affiliations:** The Second Affiliated Hospital of Guangzhou Medical University, No. 195 Dongfeng Xi Road, Guangzhou, 510182 China

**Keywords:** Glomerular mesangial cell, Proliferation, Angiotensin II, TRPC6, [Ca^2+^]^i^

## Abstract

This study investigated the changes in intracellular [Ca^2+^]^i^ (intracellular calcium ion concentration) and TRPC6 (transient receptor potential channel 6) expression during angiotensin II (AngII)-induced glomerular mesangial cell (GMC) proliferation, as well as the inhibitory effect of losartan. GMC cultures were split into four groups treated for 24 h: Group N (blank control group), Group A (10^−7 ^mol/L AngII), Group LT (10^−7 ^mol/L AngII and 10^−5 ^mol/L losartan), and Group Pred (10^−7 ^mol/L AngII and 10^−5 ^mol/L prednisone). GMCs proliferation was measured by the MTT and trypan blue assays. The distribution of TRPC6 was monitored by immunofluorescence, the expression of TRPC6 was detected by RT-PCR and Western blotting, and [Ca^2+^]^i^ was measured by laser scanning confocal microscopy. The results showed that the maximal proliferation of GMCs was induced by treatment with 10^−7 ^mol/L AngII for 24 h. In Group A, the distribution of TRPC6 was not uniform in the cell membrane, there was increased accumulation of this protein within the cytoplasm, and the increased expression of TRPC6 and [Ca^2+^]^i^ was consistent with the proliferation of cells. In Group LT, losartan inhibited the proliferation of GMCs significantly, the levels of TRPC6 and [Ca^2+^]^i^ were diminished, and the distribution of TRPC6 was improved. Prednisone also significantly inhibited the proliferation of GMCs and had no effects on the expression of TRPC6 and [Ca^2+^]^i^ in Group Pred. These findings suggested that AngII could enhance the expression of TRPC6, increase [Ca^2+^]^i,^ and demonstrate a time–dose–response relationship with the proliferation of GMCs, while losartan reversed the effect of AngII on GMC proliferation.

## Introduction

In the past 20 years, epidemiological studies have suggested an increase in the incidence of chronic kidney disease in children [1]. Glomerulosclerosis is a pathological condition commonly found in chronic kidney diseases of different causes and is characterized by glomerular mesangial cell (GMC) proliferation and extracellular matrix (ECM) deposition. Therefore, there are numerous studies regarding GMC proliferation-related factors, which are important for making progress toward the reversal of glomerular injury.

Transient receptor potential channel (TRPC) is a transmembrane protein that allows for transmembrane movement of Ca^2+^. TRPC6 is the core member of TRPC superfamily [2, 3], which maintains normal kidney function by regulating [Ca^2+^]^i^ (intracellular calcium ion concentration) and is involved in a number of kidney diseases [4, 5].Changes in intracellular calcium signaling pathways caused by mutations or abnormal expression of TRPC6 lead to various kidney proliferation diseases [6], such as focal segmental glomerulosclerosis (FSGS), minimal change disease (MCD), and membranous glomerulonephritis (MGN). These observations suggest that the relationship between TRPC6 and Ca^2+^ is of great significance for the proliferation type of kidney diseases. Angiotensin II (AngII) can activate multiple signaling pathways through the AngII type I receptor (AT1R) and is involved in the regulation of TRPC6 as well as upregulating the expression of chemotactic factor, fibroblast growth factor, and adhesion molecule, thus contributing to pathological processes such as cell proliferation, inflammation, and fibrosis [7–9]. Previous research had shown that TRPC6 can promote the increase of AngII-related Ca^2+^ through AT1R to enhance damage caused by AngII [10]. Therefore, studies of the relationship among GMC ion channels [Ca^2+^]^i^ and the involvement of ion channels in cell proliferation have become hot topics. In this study, GMC proliferation induced by AngII was observed. The expression of TRPC6 at the gene and protein level, as well as the relationship between cell proliferation and [Ca^2+^]^i^ in the GMC proliferation process, was further investigated. Furthermore, the blocking effects of losartan were studied, and the mechanism of cell proliferation induced by AngII was explored from the perspective of ion channels.

## Materials and methods

### GMC proliferation and MTT (methyl thiazolyl tetrazolium) assay

Glomerular mesangial cells (HBZY-1, Experimental Animal Center in Sun Yat-sen University, China) were cultured in incubators containing 5 % CO_2_ at 37 °C and grew adherently in 20 % fetal bovine serum medium (Gibco, USA). Morphology was observed with an inverted microscope. GMCs at logarithmic growth stage were inoculated into six-well plates, fixed with paraformaldehyde for 30 min, and then blocked by goat serum (Gibco, USA) for 1 h. Afterward, GMCs were incubated with mouse monoclonal antibodies (1:50, Wuhan Boster, China), including anti-desmin protein, anti-α-actin protein, anti-myosin protein, anti-vimentin protein, anti-keratin protein, and anti-factor VIII at 4 °C overnight. This was followed by inoculation with FITC-labeled goat anti-mouse IgG (1:32, Invitrogen, USA) at 37 °C for 1 h. GMCs were monitored and visualized by fluorescence microscopy. GMCs of 3–8 generations were used for further experiments.

Cells were inoculated into 96-well plates. In the serum-free medium, cells adhered to the surface, and different concentrations of AngII (Sigma, USA), including 0 (blank control group), 10^−8^ and 10^−7^, 10^−6^ and 10^−5 ^mol/L, were used to stimulate the GMCs for 0, 24, 48, and 72 h, respectively. Next, 10 μL of MTT (5 mg/mL, Sigma, USA) was added to each well and incubated at 37 °C for 4 h; afterward, 150 μL of dimethyl sulfoxide (DMSO) was added to each well. The optical density (OD) at 490 nm was measured by a microplate reader. After mixing the cells with a 0.4 % trypan blue solution (9:1, Wuhan Boster, China)for 3 min, the number of living cells and dead cells were counted with a hemocytometer. Proliferation rate (%) was calculated with the following formula: (OD value in AngII group − OD value in blank control group)/OD value in blank control × 100 %. Cell viability (%) was calculated with the following formula: number of living cells/(number of living cells + number of dead cells) × 100 %.

After determining the optimal concentration and time of AngII treatment (10^−7 ^mol/L of AngII to induce GMC proliferation for 24 h), the experiments were grouped as follows: Group N (blank control group), Group A (10^−7 ^mol/L AngII), Group LT (10^−7 ^mol/L AngII and 10^−5 ^mol/L losartan), and Group Pred (10^−7 ^mol/L AngII and 10^−5 ^mol/L prednisone). Intervention groups (Group LT and Group Pred) were pre-treated by losartan or prednisone, respectively, for 1 h prior to the addition of AngII. Inhibition rate (%) was calculated with the following formula: (OD value in group A − OD value in intervention group)/OD value in group A × 100 %.

### Distribution and expression of TRPC6

#### Immunofluorescence staining

Cells in different groups were fixed with paraformaldehyde, blocked with goat serum (Gibco, USA), and mixed with rabbit anti-mouse TRPC6 polyclonal antibody (1:1000, Alomone Labs, Israel). After overnight incubation, FITC-labeled goat anti-rabbit IgG (1:200, Santa Cruz, USA) was added, and the distribution of TRPC6 was observed by immunofluorescence microscopy.

#### Reverse transcription and real-time PCR

Total RNA was extracted with the addition of Trizol reagent (Invitrogen, USA) into all groups. cDNA was synthesized using 1–2 μg of total RNA. The primers were designed using the Primer Express software (Primer Premier 5.0) based on sequences obtained with the GenBank accession numbers. The sequences of the primers and amplicon sizes are shown in Table 1. PCR amplification consisted of one cycle at 95 °C for 30 s, followed by 40 cycles at 95 °C for 5 s and 60 °C for 20 s. Agarose gel electrophoresis was employed to analyze PCR products. Optical density analysis with glyceraldehyde-3-phosphate dehydrogenase (GAPDH, Invitrogen, USA) serving as an internal reference was used to measure mRNA expression in all groups.

**Table 1:** Real-time PCR primer sequences and amplicon sizes

Gene	Primer sequences (5′–3′)	Size (bp)	Accession no.
TRPC6	F: TGG CAA GTC CAG CAT ACC TGT C	178	NM 053559
(Rattus)	R: GTG TTT CTG CAG AGG TCC AGG AG		
GAPDH	F: GGC ACA GTC AAG GCT GAG AAT G	143	NM 017008
(Rattus)	R: ATG GTG GTG AAG ACG CCA GTA		

### Western blotting

Cell lysis solution was added into the cells of each group. The protein concentration was determined by the bicinchoninic acid (BCA) method. Thirty five micrograms of total protein was denatured at 100 °C for 5 min, run on a 120 g/L sodium dodecyl sulfate polyacrylamide gel (SDS-PAGE, Invitrogen, USA) for 1.5 h, and then wet transferred to a polyvinylidene difluoride (PVDF, Invitrogen, USA) membrane. The membrane was blocked with 5 % non-fat milk at room temperature for 30 min, followed by incubation with rabbit anti-mouse TRPC6 polyclonal antibody (1:1000, Alomone Labs, Israel) at 4 °C overnight. The membrane was subsequently incubated with HRP-labeled goat anti-rabbit IgG (1:5000, Wuhan Boster, China) at room temperature for 1 h. After the final rinse, the membrane was visualized with an enhanced chemiluminescence reagent and exposed to X-ray films. Band intensity was estimated using Gel-Pro image analysis software. The relative expression levels of target proteins were calculated as the ratio of the target protein band intensity to that of the internal control GAPDH.

### Intracellular [Ca^2+^]^i^

Cells from each group were re-suspended with 5 × 10^−6 ^mol/L of Fluo-3AM (Invitrogen, USA) at 37 °C for 45 min. Following this step, cells were inoculated into special culturing dishes for laser scanning confocal microscope (LSCM) and washed with D-Hanks three times. Then, the measurements were taken by LSCM with the excitation wavelength set at 488 nm. The [Ca^2+^]^i^ was scanned per 5 s for 10 min, with each scan covering 20 cells. The results were analyzed by Zeiss image 4.2 software. The [Ca^2+^]^i^ was calculated with the following formula: F/Fa (F: real-time fluorescence intensity; Fa: basic fluorescence intensity).

### Statistical analyses

All values were expressed as mean ± SD, and analysis of variance was calculated using SPSS 11.5 statistical analysis software. Fisher’s LSD test was used for the pairwise comparison. A *P* value <0.05 was considered to be statistically significant.

## Results

### Morphology and identification of GMC

**Fig. 1:**
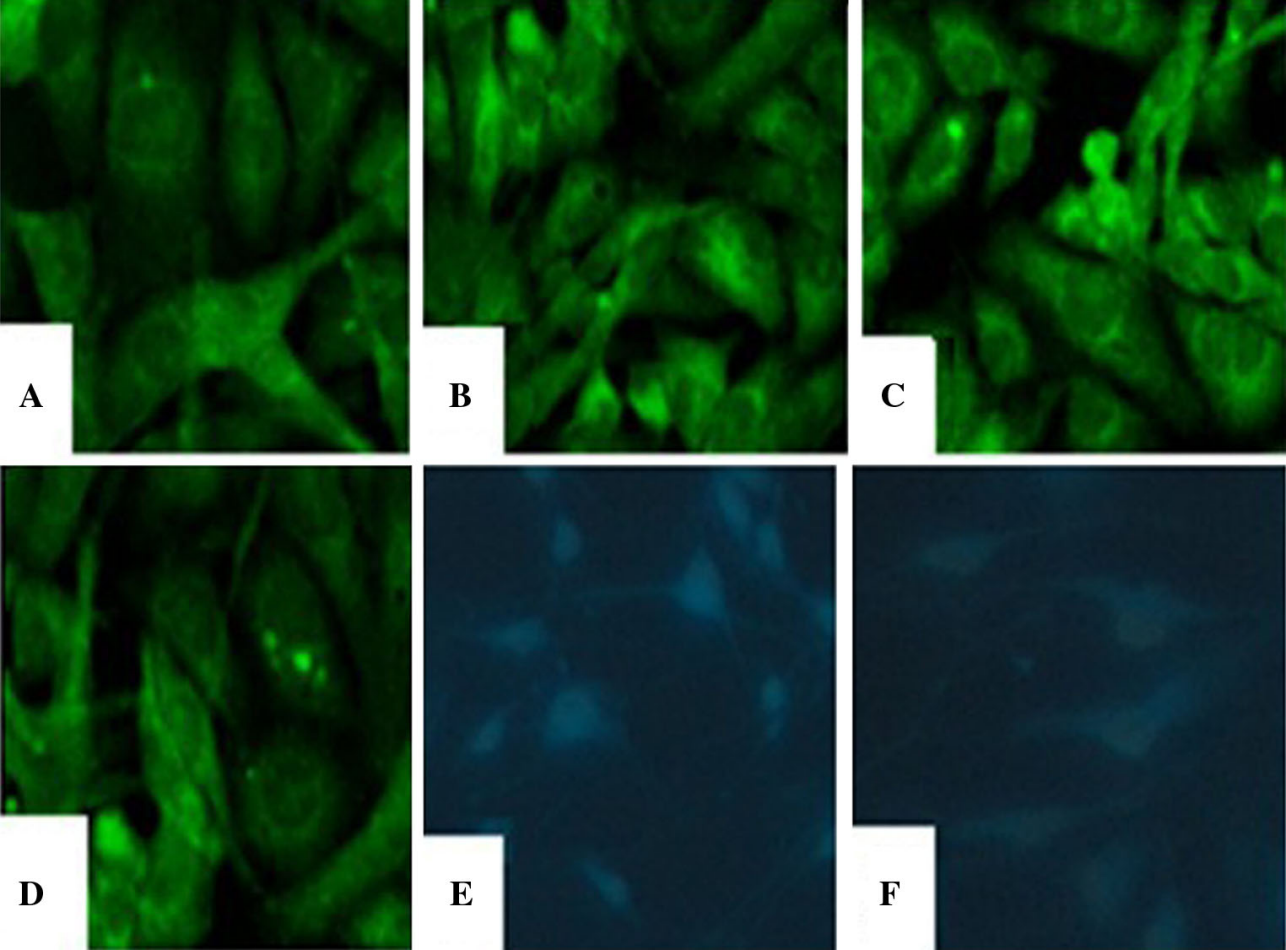
Images of immunofluorescence staining for GMC (×400). **a** Desmin(+); **b** a-Actin(+); **c** Myosin(+); **d** Vimentin(+); **e** Keratin(−); **f** Factor VIII (−) (*Blue color* was DAPI nuclei staining, the target protein(−)) (color figure online)

GMCs grew adherently and showed larger soma with fusiform, irregular star-shaped, or elongated shapes. There were several dendrites of various lengths on the cell, which connected the cell groups into a network with a round- or egg-shaped cell nuclei in the middle. The GMCs stained positive for desmin, α-Actin, myosin, and vimentin and negative for keratin and factor VIII, indicating that the cultured cells were in fact GMCs (Fig. 1).

### Cell proliferation results

There was no obvious cell proliferation induced by the addition of 10^−8 ^mol/L AngII, while addition of 10^−7^, 10^−6,^ and 10^−5 ^mol/L of AngII caused increased cell proliferation at 24, 48, and 72 h; the proliferation at 24 h was much more significant than that at 48 h and 72 h (*P* < 0.01, *P* < 0.05). In addition, at 24 h, the proliferation with AngII at 10^−7^ mol/L was of greater statistical significance than that observed with AngII at 10^−8^, 10^−6,^ and 10^−5^ mol/L (*P* < 0.01) (Table 2). After staining with trypan blue, dead cells turned into the color of light blue while living cells kept unchanged in color. The cell viability for cells treated with 10^−7^ mol/L AngII at 24 h was 91–95 %, while cell viabilities of 80–86 and 60–68 % were observed when cells were treated with 10^−6^ and 10^−5^ mol/L of AngII, respectively. Compared with Group N, the increases in GMC proliferation in Group A, Group LT, and Group Pred were statistically significant (*P* < 0.01, *P* < 0.05). Furthermore, compared with Group A, the GMC proliferation was significantly inhibited in Group LT and Group Pred (39.5 and 49.6 %, *P* < 0.05) (Table 3).

**Table 2:** Changes in GMC proliferation induced by AngII (mean ± SD)

Groups (mol/L)	0 h	24 h	48 h	72 h
0	0.147 ± 0.016	0.230 ± 0.021	0.161 ± 0.020	0.162 ± 0.017
10^−8^	0.149 ± 0.013	0.298 ± 0.014 (29.6 %)	0.217 ± 0.018 (34.4 %)	0.202 ± 0.015 (92.3 %)
10^−7^	0.152 ± 0.027	0.699 ± 0.012 (203.6 %)**^##△△abc^	0.349 ± 0.025 (116.1 %)**	0.470 ± 0.016 (189.8 %)**
10^−6^	0.152 ± 0.018	0.411 ± 0.021 (78.6 %)*^#△^	0.330 ± 0.022 (104.4 %)*	0.291 ± 0.019 (79.6 %)*
10^−5^	0.151 ± 0.022	0.421 ± 0.019 (83.1 %)*^#△^	0.338 ± 0.019 (109.7 %)*	0.281 ± 0.018 (73.5 %)*

(%) Proliferation rate; versus Group 0 mol/L, * *P* < 0.05, ** *P* < 0.01; Group 24 h versus 48 h, ^#^ *P* < 0.05, ^##^ *P* < 0.01; Group 24 h versus 72 h, ^△^ *P* < 0.05, ^△△^ *P* < 0.01; Group 24 h 10^−7^ versus 24 h 10^−8^

^a^ *P* < 0.01; Group 24 h 10^−7^ versus 24 h 10^−6^, ^b^ *P* < 0.01; Group 24 h 10^−7^ versus 24 h 10^−5^, ^c^ *P* < 0.01

**Table 3:** Inhibitory effects of losartan and prednisone on GMC proliferation (mean ± SD)

Groups	24 h	Inhibition rate (%)
N	0.224 ± 0.020	
A (AngII 10^−7^ mol/L)	0.691 ± 0.019**	
LT (AngII 10^−7^ mol/L + LT 10^−5^ mol/L)	0.418 ± 0.021*^#^	39.5 %
Pred (AngII 10^−7^ mol/L + Pred^−5^ mol/L)	0.348 ± 0.018*^#^	49.6 %

Versus Group N, * *P* < 0.05, ** *P* < 0.01; versus Group A, ^#^ *P* < 0.05

### TRPC6 expression

#### TRPC6 distribution

**Fig. 2:**
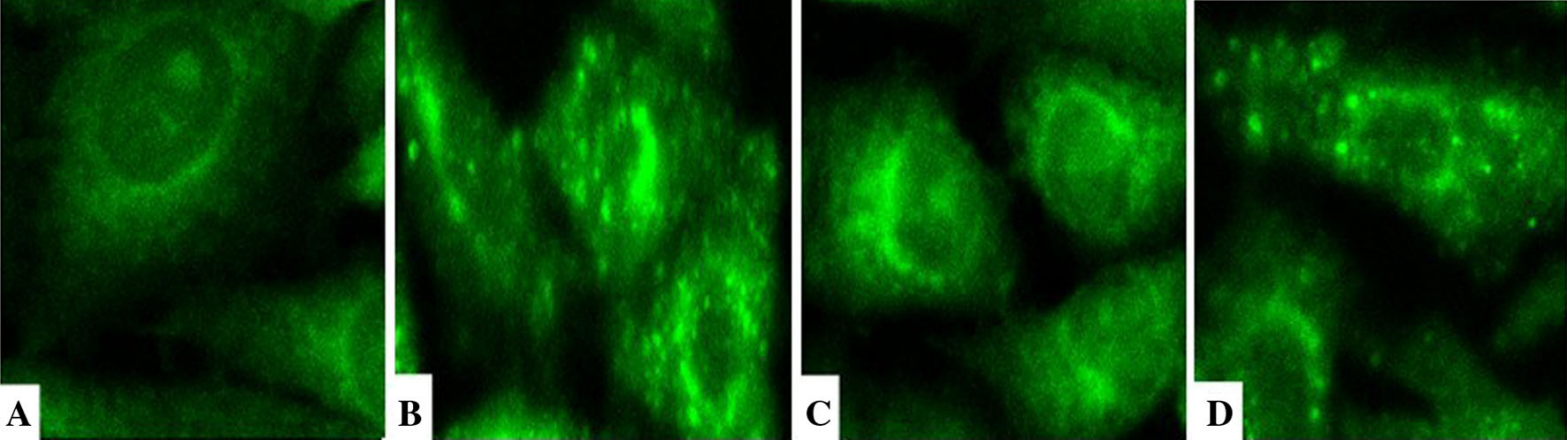
Distribution images of TRPC6 in GMC (×600). **a** Group N: TRPC6 was uniformly distributed in cell membrane and cytoplasm, most in cell membrane and few in cytoplasm; **b** Group A: TRPC6 distribution was not continuous along membrane, with some membrane region missing of TPRC6; more TRPC6 appeared in cytoplasm, even particles can be observed; **c** Group LT: TRPC6 distribution was more uniform and improved than that in group A; **d** Group Pred: TRPC6 distribution was similar as in group A, with some regions along cell membrane missing and more in cytoplasm

In Group N, TRPC6 was detected mostly in the cell membrane, where it was uniformly distributed and showed little staining in the cytoplasm (Fig. 2a). After introduction of AngII for 24 h (Group A), the distribution of TRPC6 was no longer homogeneous in the membrane, and some region showed a lack of TPRC6 staining; additionally, more TRPC6 was detected in cytoplasm, and even particles were observed (Fig. 2b). Compared with Group A, after treatment by losartan (Group LT), the distribution of TRPC6 was more uniform and improved (Fig. 2c). After the incubation with prednisone (Group Pred), the distribution of TRPC6 was similar to that observed in Group A, with some regions along cell membrane missing TPRC6 and more appearing in cytoplasm (Fig. 2d).

#### TRPC6 mRNA expression

**Fig. 3:**
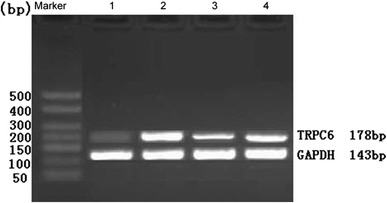
Expression of GMC TRPC6 mRNA in different groups. *1* Group N; *2* Group A; *3* Group LT; *4* Group Pred

The expression of TRPC6 mRNA and GAPDH mRNA was measured (Fig. 3). Compared with Group N, the expression of TRPC6 at the mRNA level in Group A, Group LT, and Group Pred was elevated (*P* < 0.01); when compared with Group A, the expression of TRPC6 mRNA in Group LT was diminished (*P* < 0.01), and there existed no significant difference in Group Pred (*P* > 0.05) (Fig. 5).

#### TRPC6 protein expression

**Fig. 4:**
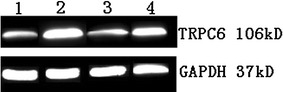
Electrophoresis for TRPC6 protein of GMC in different groups. *1* Group N; *2* Group A; *3* Group LT; *4* Group Pred

**Fig. 5:**
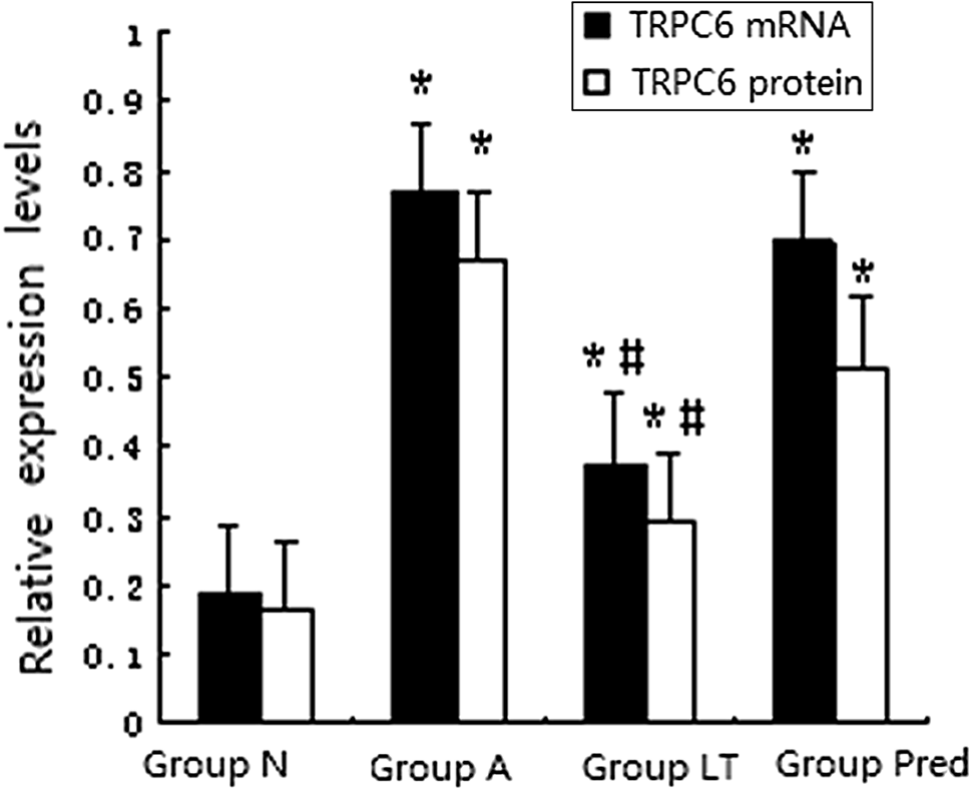
The analysis of TRPC6 mRNA and protein of GMC in different groups. Versus Group N, *P < 0.01; versus Group A, ^#^ *P* < 0.01

The proteins measured by immunoblotting were the 106 kDa TRPC6 protein and the 37 kDa GAPDH protein (Fig. 4). The expression of the TRPC6 protein in Group A, Group LT, and Group Pred was higher than that in Group N (*P* < 0.01); as compared with Group A, the expression of TRPC6 protein in Group LT declined (*P* < 0.01), and no significant difference in Group Pred was observed (*P* > 0.05) (Fig. 5).

### Changes of [Ca^2+^]^i^

**Fig. 6:**
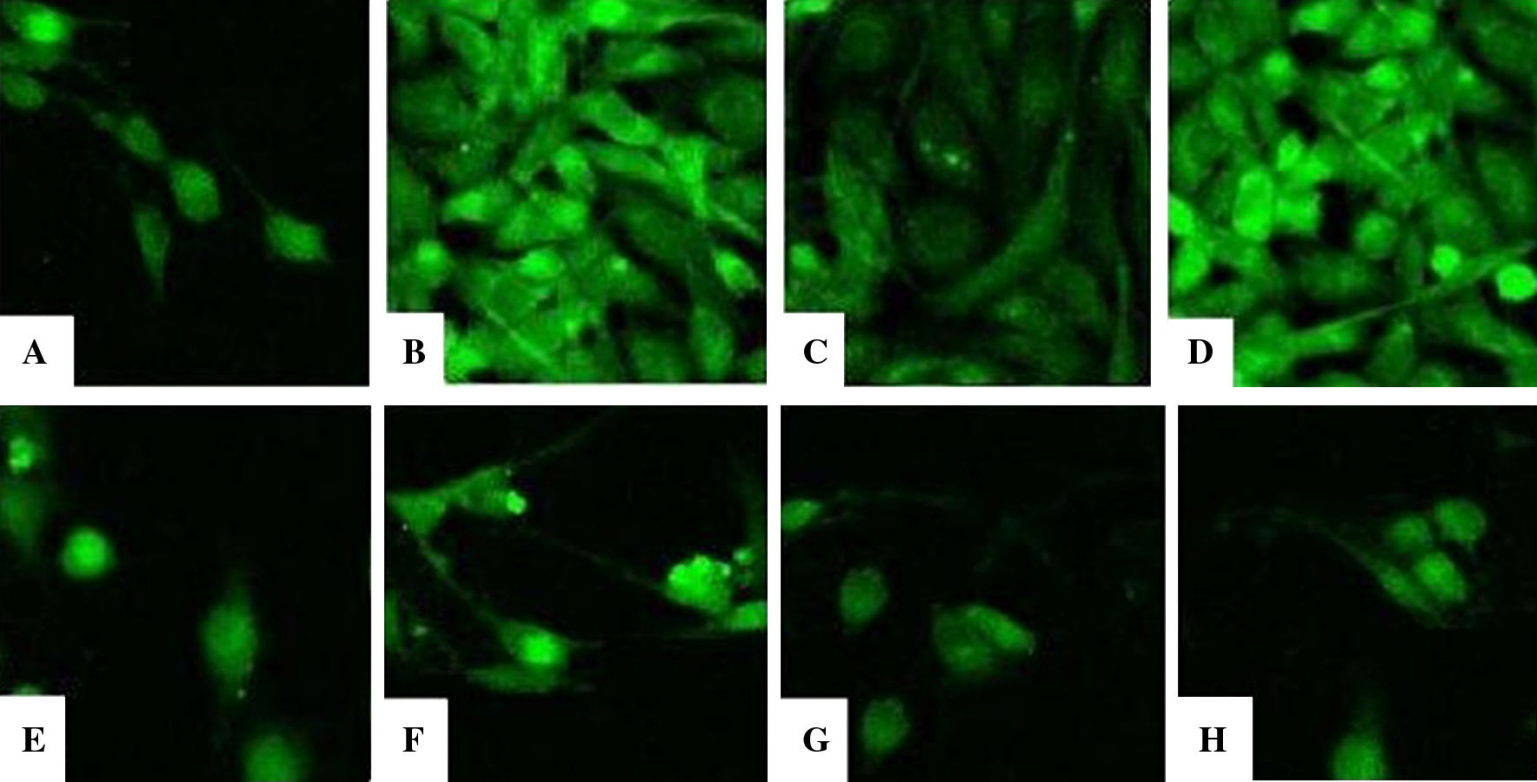
Distribution of [Ca^2+^]^i^ for GMC at 4 min and 8 min (×400). **a** Group N at 4 min; **b** Group A at 4 min; **c** Group LT at 4 min; **d** Group Pred at 4 min; **e** Group N at 8 min; **f** Group A at 8 min; **g** Group LT at 8 min; **h** Group Pred at 8 min

**Fig. 7:**
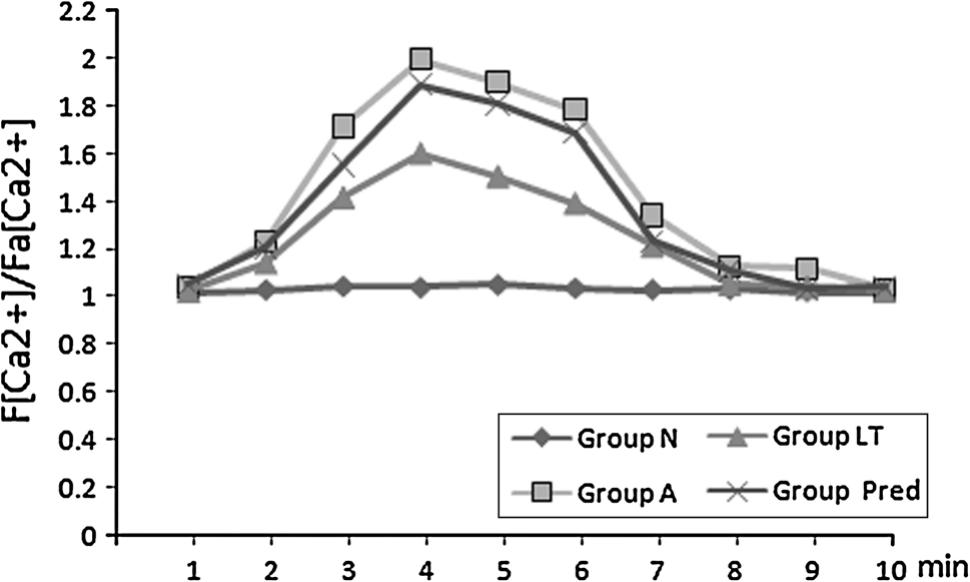
Changes of [Ca^2+^]^i^ in GMC in different groups

After incubation with Fluo-3AM, a calcium indicator, green fluorescence was uniformly distributed in cells (Fig. 6). The [Ca^2+^]^i^ increased significantly after the addition of AngII. The fluorescence intensity began to increase at the third min and peaked at the fourth minute. The fluorescence began to decrease 1 min later and returned to the starting level at the eighth minute (Fig. 7). At the fourth min, [Ca^2+^]^i^ in Group A, Group LT, and Group Pred were higher than that in Group N (*P* < 0.01); as compared with Group A, [Ca^2+^]^i^ decreased in Group LT (*P* < 0.05), and there was no significant difference in Group Pred (*P* > 0.05).

## Discussion

The characteristic pathological features of early-stage glomerulosclerosis are excessive proliferation of GMCs and secretion of large amounts of ECM in the glomerulus, which eventually leads to glomerulosclerosis-induced renal failure. As the main active substances in the kidney, AngII can enhance the mRNA and protein expression of transforming growth factor-beta and fibrinolytic enzyme activator inhibitor, causing GMC proliferation and ECM accumulation that gradually leads to glomerulosclerosis.

In this study, the cell proliferation was indirectly measured by the methyl thiazolyl tetrazolium assay, which detected the activity of mitochondrial metabolic enzymes, in order to measure AngII-induced GMC proliferation. The results showed that at concentrations of 10^−7^, 10^−6,^ and 10^−5 ^mol/L, AngII promoted the proliferation of GMCs during the 0–48 h time frame after treatment; there was an apparent time–dose–response relationship, especially for 10^−7^ mol/L AngII at 24 h. When the concentration of AngII was increased to 10^−5^ mol/L, which was at 72 h, the cell proliferation delayed, which indicated that AngII affected not only cell proliferation, but also cell apoptosis. AngII receptors mainly include AT1R and AT2R; AT1R mainly affects cell proliferation and smooth muscle contraction, while AT2R affects cell apoptosis and smooth muscle relaxation [11]. Within certain concentrations of AngII, AT1R in GMCs plays the leading role, so the effect of AngII is mainly cell proliferation. At higher concentrations, where the number of AngII exceeds the number of AT1R binding sites, the function of AT2R comes into play. In this study, when compared with Group Pred, losartan significantly inhibited proliferation of GMCs, which suggested that by blocking the binding between AngII and AT1R, cell proliferation could be effectively inhibited.

Intracellular Ca^2+^ is as an important second messenger in the process of cell proliferation, and the changes in concentration trigger the transduction of growth-related signals. An increase of [Ca^2+^]^i^ can promote the proliferation of GMCs; for example, AngII can increase [Ca^2+^]^i^ by signal transduction pathways, which in turn regulates cell proliferation [12, 13]. After treatment with a calcium chelator, the increase of [Ca^2+^]^i^ and the proliferation of GMCs are significantly inhibited [14]. The same inhibitory effect was shown for losartan in this study. Store-operated calcium channels (SOCC) participate in signal transduction as major regulators of Ca^2+^ internal flow in kidney micro-vascular smooth muscle cells and GMCs. TRPC1/3/4/6 of GMCs are candidates for SOCC [15]. Through SOCC and other types of calcium channels, AngII can affect intracellular Ca^2+^, thereby regulating renal microcirculation and GMC functions [16].

In this study, it was observed that AngII not only stimulated the proliferation of GMCs, but also significantly affected the distribution of TRPC6 and its expression at the mRNA and protein level. Measurements with a confocal microscope indicated that [Ca^2+^]^i^ reached the maximum level at the forth minute after incubation with AngII. These observations suggested that TRPC6 and Ca^2+^ played important roles in the process of GMC proliferation. After incubation with losartan, TRPC6 distribution was improved, and [Ca^2+^]^i^ was consistent with the cell proliferation, indicating that AngII affected TRPC6 and Ca^2+^ through AT1R, and further regulated cell proliferation. Prednisone inhibited cell proliferation, but had no effect on the expression of TRPC6 and [Ca^2+^]^i^, indicating that cell proliferation was not necessarily caused by changes in [Ca^2+^]^i^. It had been reported that glucocorticoid does not cause the changes in [Ca^2+^]^i^, and that its effects on cell proliferation could be achieved by inhibiting nuclei factor NF-κB or increasing P21 protein to arrest the cell cycle of GMCs at G_0_/G_1_ [17, 18].

AngII promoted the increase of [Ca^2+^]^i^ in GMCs, which in turn induced its proliferation. The mechanism by which this occurs begins with the binding of AngII to AT1R on the surface of GMCs, which causes phospholipase c (PLC) to catalyze the cleavage of 4,5-phosphatidyl inositol diphosphate (PIP2), producing 1,4,5-inositol triphosphate (IP3). IP3 can then activate Ca^2+^ channels, leading the flow of extracellular Ca^2+^ into the cell. This influx results in increased [Ca^2+^]^i^ in the sarcoplasm, which in turn regulates transcription and causes the proliferation of GMCs. There exist target sites for AngII on the TRPC6 channel. Through TRPC6, [Ca^2+^]^i^ can be increased by AngII stimulation. And it is possible that TRPC6 and AngII are relevant in terms of regulating GMC proliferation via controlling [Ca^2+^]^i^ [19]. In the case of receptor-regulating TRPC6 signaling pathways, high IP3 causes the release of sensitive calcium stores that further activate TRPC6, which leads an influx of Ca^2+^ into the cells to fill the calcium stores, so TRPC6 is considered a candidate for SOCC and plays the role of a SOCC [20]. These studies suggested that the mechanism by which AngII promoted the increase of [Ca^2+^]^i^ and induced GMC proliferation shared similarities with the mechanism by which TRPC6 regulated [Ca^2+^]^i^, as both were associated with an IP3-regulated calcium channel, and TRPC6 mediated Ca^2+^ to flow inward to promote the proliferation of GMCs.
